# Unveiling the operation mechanism of layered perovskite solar cells

**DOI:** 10.1038/s41467-019-08958-9

**Published:** 2019-03-01

**Authors:** Yun Lin, Yanjun Fang, Jingjing Zhao, Yuchuan Shao, Samuel J. Stuard, Masrur Morshed Nahid, Harald Ade, Qi Wang, Jeffrey E. Shield, Ninghao Zhou, Andrew M. Moran, Jinsong Huang

**Affiliations:** 1 Department of Mechanical and Materials Engineering, University of Nebraska-Lincoln, Lincoln, NE 68588 USA; 2 Department of Applied Physical Sciences, University of North Carolina, Chapel Hill, NC 27599 USA; 3 Department of Physics, North Carolina State University, Raleigh, NC 27695 USA; 4 Nebraska Center for Materials and Nanoscience, University of Nebraska-Lincoln, Nebraska, 68588 USA; 5 Department of Chemistry, University of North Carolina, Chapel Hill, NC 27599 USA

## Abstract

Layered perovskites have been shown to improve the stability of perovskite solar cells while its operation mechanism remains unclear. Here we investigate the process for the conversion of light to electrical current in high performance layered perovskite solar cells by examining its real morphology. The layered perovskite films in this study are found to be a mixture of layered and three dimensional (3D)-like phases with phase separations at micrometer and nanometer scale in both vertical and lateral directions. This phase separation is explained by the surface initiated crystallization process and the competition of the crystallization between 3D-like and layered perovskites. We further propose that the working mechanisms of the layered perovskite solar cells involve energy transfer from layered to 3D-like perovskite network. The impact of morphology on efficiency and stability of the hot-cast layered perovskite solar cells are also discussed to provide guidelines for the future improvement.

## Abstract (web summary)

It is well-accepted that the two dimensional layered halide perovskite can improve the device stability of perovskite solar cells but the operation mechanism remains unclear. Here Lin et al. reveal the real morphology of the hot-cast layered perovskite solar cells and understand the working mechanism.

## Introduction

Organic–inorganic hybrid perovskites (OIHPs) have emerged as the most promising low-temperature solution-processable photovoltaic materials due to their intriguing properties like long carrier diffusion length, large absorption coefficient and low bulk trap density^1–3^. Despite the significant improvement in power conversion efficiency (PCE) achieved in the past few years^4^, the concern on the poor stability, especially the moisture stability, of OIHP materials remains a major hurdle for the commercialization of the OIHP solar cells^5^. The low stability stems mainly from the low formation energy and hydroscopic nature of the OIHP materials^6^. One approach to address the poor moisture stability of three-dimensional (3D) perovskite devices is to introduce layered OIHPs, or quasi-two-dimensional (2D) perovskites, which contain sheets of 3D perovskites sandwiched by long organic ligands. The hydrophobic ligands can slow down the permeation of moisture into the lead iodide octahedron, and thus impede the decomposition of the OIHPs by moisture^7,8^. Since the photovoltaic devices fabricated by layered perovskite materials exhibit impressively better stability in some cases, these materials have recently attracted intensive attention, despite that their PCEs are still much lower than the 3D perovskite counterparts^7–10^.

The further PCE improvement of layered perovskite solar cells relies on better understanding of their operation mechanism, which, however, remains largely elusive up to now. Tsai et al.^9^ reported that the layered perovskite film fabricated with hot-casting spin-coating method showed the preferential alignment of the layered crystallographic planes along the out-of-plane direction, and assigned the high mobility in the crystallographic planes of the inorganic perovskite components is the main reason for the efficient charge carrier extraction and thus high PCE above 12% in layered perovskite solar cells. Nevertheless, the underlying working mechanism for solar cells involving layered perovskites is still not clear. For example, the morphology, phase compositions and distribution are not known yet for the perovskite thin films prepared with a composition for layered perovskites. Liu et al.^11^ observed very different photoluminescence (PL) properties from the top and bottom film surface, which was explained by different layered perovskite phases with increasing *n* values naturally aligned along the vertical direction to the substrate. In addition, it is still an open question of how the photogenerated excitons efficiently dissociate in layered perovskites with exciton binding energy much larger than thermal energy at room temperature^12–14^. Blancon et al.^15^ proposed that the low energy edge states in layered perovskites can facilitate exciton dissociation. However, a full picture of exciton diffusion to edge states and free charge carrier extraction is still missing.

For a better understanding of the working principle of layered perovskite solar cells, in this study, we carefully examine the morphology of the hot-cast layered perovskite thin films at both microscopic and nanoscopic scales. The results show that layered perovskite thin films comprise multiple layered perovskite phases surrounded by 3D-like perovskites. The layered flakes are much smaller than the thin film thickness. Based on the observed morphology, we propose a model for the operation of realistic layered perovskite solar cells which involves energy transfer from layered to 3D-like perovskites and charge collection through the 3D-like perovskite network.

## Results

### Material preparation and device evaluation

**Fig. 1:**
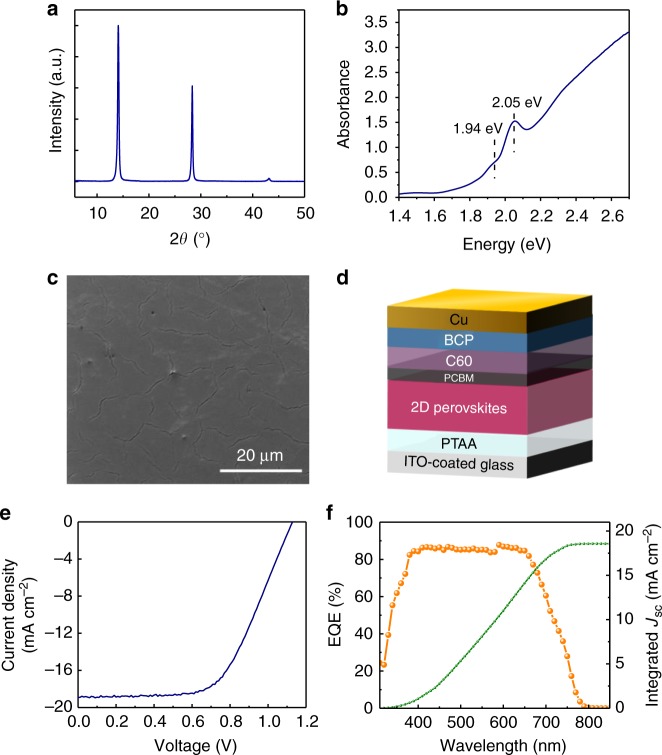
Characterization of layered perovskite thin films and fabricated solar cells. X-ray diffraction (XRD) spectrum (**a**), absorption spectrum (**b**) and scanning electron microscopy (SEM) image (**c**) of layered perovskites films. **d** The layered perovskite photovoltaic device architecture. **e** Current density-voltage (*J-V*) curve of solar cells based on layered perovskites. **f** External quantum efficiency (EQE; orange) and integrated short-circuit current density (*J*_SC_; green) as a function of wavelength for layered perovskite solar cells

Achieving efficient layered perovskite solar cells is essential to establish the material platform for investigating the working mechanism. In this study, layered perovskite films with a nominal composition of (BA)_2_(MA)_3_Pb_4_I_13_ (BA = CH_3_(CH_2_)_3_NH_3_^+^; MA = CH_3_NH_3_^+^) were fabricated by hot-casting method. The precursor solutions were prepared by dissolving specific stoichiometric quantities of *n*-butylammonium iodide (BAI), methylammonium iodide (MAI) and PbI_2_ in *N,N*-dimethylformamide (DMF). The X-ray diffraction (XRD) pattern of layered perovskite films is shown in Fig. 1a. Three diffraction peaks at 14.20°, 28.48° and 43.28° were well resolved, which match the diffraction peak positions of ($\bar 1$1$\bar 1$), (202) and (313) crystallographic planes from layered perovskite (BA)_2_(MA)_3_Pb_4_I_13_, respectively^9^. However, the diffraction peaks of (110), (220) and (314) crystallographic planes from 3D perovskite MAPbI_3_ located at 14.05°, 28.45° and 43.13° or ($\bar 1$1$\bar 1$), (202) and (313) crystallographic planes from layered perovskite (BA)_2_(MA)_*n*-1_Pb_*n*_I_3*n*+1_ (*n* = 2, 3) also agreed with the XRD pattern quite well^16–18^. The calculated diffraction angle 2*θ* values of layered perovskites (*n* = 2 to 4) were summarized (Supplementary Table 1, Supplementary Note 2). The difference of 2*θ* values between the same crystallographic planes of (BA)_2_(MA)_*n*-1_Pb_*n*_I_3*n*+1_ (*n* = 2 to 4) are within 0.12°, which are negligible compared to the FWHM (full width at half maximum) of the diffraction peaks (greater than 0.45° for the XRD result presented here), making it even more difficult to index the different perovskite phases. There are not clear peaks at diffraction angles (2*θ*) below 14° which are characteristic of low-angle {010} reflections in layered crystals^18^. In order to further determine the phase composition, XRD measurements of scraped powder of thin films were also conducted. Several extra diffraction peaks are present in powder XRD pattern (Supplementary Figure 1). After comparing it with the XRD patterns of 3D perovskite powders, we concluded that these high-angle diffraction peaks more likely originate from 3D perovskites, indicating the coexistence of layered and 3D-like perovskite phases in layered perovskite films. Weak diffraction peaks at low-angle (less than 14°) can be observed in the powder XRD spectrum; the absence of such peaks in layered perovskite thin film samples might be explained by the small amount of randomly oriented layered perovskite phases present. Exciton-like absorption peaks at 1.94 eV (*n* = 4) and 2.05 eV (*n* = 3) in Fig. 1b indicate the films prepared from mixed precursor solution contain layered perovskites^11^. The blue-shift of absorption in the long wavelength region from 3D-like perovskite phase may be ascribed to quantum confinement effect of small perovskite crystallites produced by bulking amine ligands effect, and similar results have been reported elsewhere^19,20^. As the scanning electron microscopy (SEM) image shown in Fig. 1c reveals, uniform, pinhole-free and compact layered perovskite films were obtained by the hot-casting technique. The photovoltaic devices were fabricated with a structure of indium tin oxide (ITO)/poly(bis(4-phenyl)(2,4,6-trimethylphenyl)amine) (PTAA)/layered perovskites/[6,6]-phenyl-C61-butyric acid methyl ester (PCBM)/C_60_/2,9-dimethyl-4,7-diphenyl-1,10-phenanthroline (BCP)/Cu as shown in Fig. 1d. A reasonably high PCE of 12.7% was recorded from current density-voltage (*J*-*V*) scanning, as shown in Fig. 1e, with an open-circuit voltage (*V*_OC_) of 1.13 V, short-circuit current density (*J*_SC_) of 18.90 mA cm^−2^ and fill factor (FF) of 59%. The external quantum efficiency (EQE) and integrated *J*_SC_ are shown in Fig. 1f. The integrated *J*_SC_ of 18.57 mA cm^−2^ agrees well with the measured *J*_SC_ from *J*-*V* scanning. The layered perovskite thin films used for the morphology investigation in this study were fabricated with the same method as those in our high PCE layered perovskite solar cells.

**Fig. 2:**
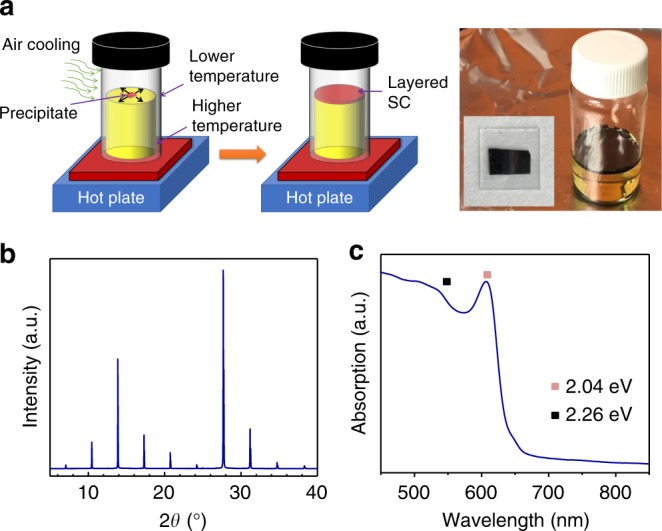
Layered perovskite single crystals synthesis and characterization. **a** (left panel) Schematic of (BA)_2_(MA)_2_Pb_3_I_10_ single crystal plate growth method; (right panel) photograph of (BA)_2_(MA)_2_Pb_3_I_10_ single crystal platelet floating on the solution surfaces in a glass vial (the vial diameter is 25 mm), and the hand-cut rectangular single crystal platelet stick to a glass substrate (15 mm × 15 mm). X-ray diffraction (XRD) pattern (**b**) and absorption spectra (**c**) of (BA)_2_(MA)_2_Pb_3_I_10_ single crystal (the pink dot indicates the main exciton peak and the black dot indicates the position of the band-to-band absorption edge)

We also synthesized layered perovskite single crystals for anisotropic mobility studies. The layered perovskite single crystals were synthesized following the liquid phase crystallization method (Fig. 2a, left panel). Typically, for layered (BA)_2_(MA)_2_Pb_3_I_10_ single crystal with *n* = 3, lead(II) iodide, *n*-butylamine and methylammonium iodide in appropriate molar ratios were dissolved in hydriodic acid solvent by heating. Subsequently, the hot solution was slowly cooled to room temperature and layered perovskite (BA)_2_(MA)_2_Pb_3_I_10_ single crystal platelets can be obtained at the solution–air interface (Fig. 2a, right panel). The XRD pattern (Fig. 2b) revealed three low-angle diffraction peaks between 2*θ* = 5 and 14°, which were indexed to be (040), (060) and (080) crystallographic planes of layered perovskite (BA)_2_(MA)_2_Pb_3_I_10_ (*n* = 3). The calculated *d*-spacing matches the distance between the discrete perovskite layers in (BA)_2_(MA)_2_Pb_3_I_10_ {010} reflection (Supplementary Table 2, also see Supplementary Note 2)^18^. The optical absorption properties of the mechanically exfoliated layered perovskite single crystals (*n* = 3) are shown in Fig. 2c. The main exciton peak position, the onset of band-to-band absorption and the estimated exciton binding energy agree with previously reported results^15^.

### Morphology of hot-cast layered perovskite thin films

The features of the absorption spectra of the hot-cast layered perovskite thin films indicate the presence of multiple phases of layered perovskites. The results from grazing-incidence wide-angle X-ray scattering (GIWAXS) show further evidence of multiple phases and/or orientations (Supplementary Figure 2, Supplementary Figure 3 and Supplementary Note 1). As an exercise, the indexing of peaks from both layered perovskites (*n* = 4) and 3D-like perovskite phases suggest that both layered and 3D-like perovskites could be present at certain orientations in the samples. Furthermore, as indicated by the few unindexed peaks, other phases/orientations are likely present as well besides these specific layered (*n* = 4) and 3D-like phases. To obtain an in-depth understanding of the morphology of the layered perovskite thin films, we investigated the distribution, size and orientation of the layered perovskite phases using a combination of nanoscopic- and microscopic-level morphology characterizations.

### Vertical phase segregation

**Fig. 3:**
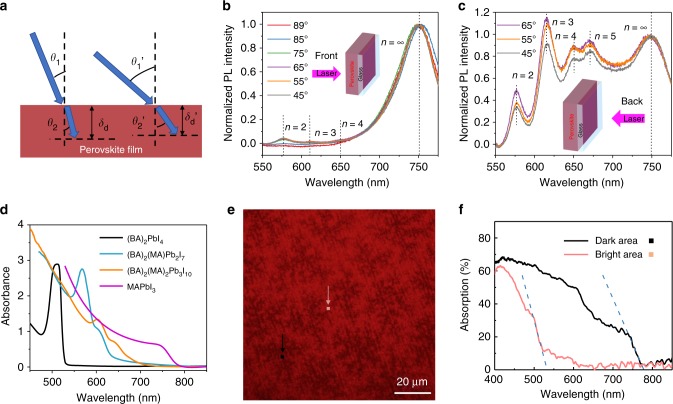
Vertical and lateral phase segregation investigation. **a** Schematic of incident-angle dependent photoluminescence (PL) setup. **b** The incident-angle-dependent PL spectra of the layered perovskite films illuminated from the front sides (as illustrated in the inset) of the films. **c** The incident-angle-dependent PL spectra of the layered perovskite films illuminated from the back sides (as illustrated in the inset) of the films. **d** Absorption spectra of three-dimensional (3D) MAPbI_3_ thin films and the layered (BA)_2_(MA)_*n*-1_Pb_*n*_I_3*n*+1_ perovskite thin films (*n* = 1 to 3). **e** Optical mapping of the layered perovskite (BA)_2_MA_3_Pb_4_I_13_ films using an optical microscope in transmission mode. Typical bright and dark area are labeled with pink and black squares, respectively. **f** Absorption spectra measured in the bright and dark area labeled in **e**. The size of the measured area is 0.6 × 0.6 µm^2^, smaller than the labeled area

First, the incident-angle-dependent photoluminescence was adopted to investigate the distribution of layered perovskite phases along the out-of-plane direction. Here, by changing the incident angle, we can control the penetration depth of the excitation laser into perovskite films (Fig. 3a). The PL measurement was conducted under two different excitation configurations, i.e., the incident laser beam with wavelength of 405 nm illuminated the perovskite films either from the air (front) side or the glass (back) side, as illustrated in the insets of Fig. 3b and Fig. 3c, and the corresponding PL spectra are shown in Fig. 3b and Fig. 3c, respectively. For both front and back excitations, the PL spectra show several emission peaks, agreeing with the absorption spectra results and GIWAXS indications that the layered perovskite films comprise multiple perovskite phases and orientations. Five PL peaks at 576 nm, 615 nm, 650 nm, 673 nm and 750 nm were observed. The PL peak at 750 nm can be assigned to 3D-like perovskites^15^. The blue-shift of this peak compared to the PL peak of pure 3D perovskites which regularly locate at a position of 780 nm may also be ascribed to the quantum confinement effect. We observed that the film thickness has an obvious effect on this peak position, which shows a blue-shift with the reducing film thickness, as shown in Supplementary Figure 4. After comparing these PL peaks with previous reports, the higher energy PL peaks at 576 nm, 615 nm, 650 nm and 673 nm can be assigned to emission from (BA)_2_(MA)_*n*-1_Pb_*n*_I_3*n*+1_ perovskites with *n* = 2, 3, 4 and 5, respectively^11^. The PL intensity had been normalized according to the intensity of the 750 nm emission peak.

The PL intensity ratio between different emission peaks obtained with different incident angles suggested that the distribution of different phases of layered perovskites was not uniform along the out-of-plane direction in the films. Under front excitation with incident light angle (the angle between the incident ray and the normal direction) of 89°, the incident laser at wavelength of 405 nm has the smallest penetration depth of 57.7 nm (the estimation of penetration depths was summarized in Supplementary Table 3). PL intensity of the dominant emission peak at 750 nm is 10 times stronger than those of 3 other emission peaks. With decreasing the incident angle and increasing penetration depth of excitation light, PL intensities from perovskite phases with *n* = 2, 3 and 4 gradually increase. Under back excitation with penetration of 64.9 nm, PL intensities of the emission from perovskite phases with *n* = 2 to 5 are comparable to that of perovskite phases with *n* ≈ ∞. The difference of PL spectra between front excitation and back excitation and different excitation depths imply that the layered perovskite phases with smaller *n* prefer to stay at the bottom, while the large *n* layered or 3D-like perovskite phases tend to stay close to the surface of the films. This is consistent with the previous study by Liu et al.^11^. The film thickness could have an effect on such graded distribution along vertical direction reported in previous work, and thicker films exhibit more dramatic difference in phase distribution throughout the sample thickness (as shown in Supplementary Figure 4)^21^.

### Lateral phase segregation

After identifying the nonuniform distributions of layered perovskite and 3D-like perovskite phases in vertical direction, we conducted the optical transmission mapping measurement to study their lateral distributions by leveraging the differences between the absorption spectra of layered and 3D perovskites. As shown in Fig. 3d, 3D perovskite has much stronger absorption than layered perovskites in the wavelength range between 680 nm and 770 nm. In the transmission mode, the incident light with wavelength less than 650 nm will be filtered after applying a 650 nm long pass filter. Therefore, the area with more layered perovskite phases appear brighter as less incoming light can be absorbed. In contrast, the area with more 3D perovskite phase appears darker in the transmission mapping images. For the hot-cast layered perovskite thin films, dendrite structure with the size in the range of several to tens of micrometers were observed, indicating the nonuniform distribution of different perovskite phases in the lateral direction (Fig. 3e). By further comparing the absorption spectra of the dark and bright area (Fig. 3f), we conclude that the dark areas correspond to 3D-like perovskite phase while the bright areas correspond to layered perovskite phases, and layered perovskites was surrounded by 3D-like perovskites.

### Morphology at the atomic scale

**Fig. 4:**
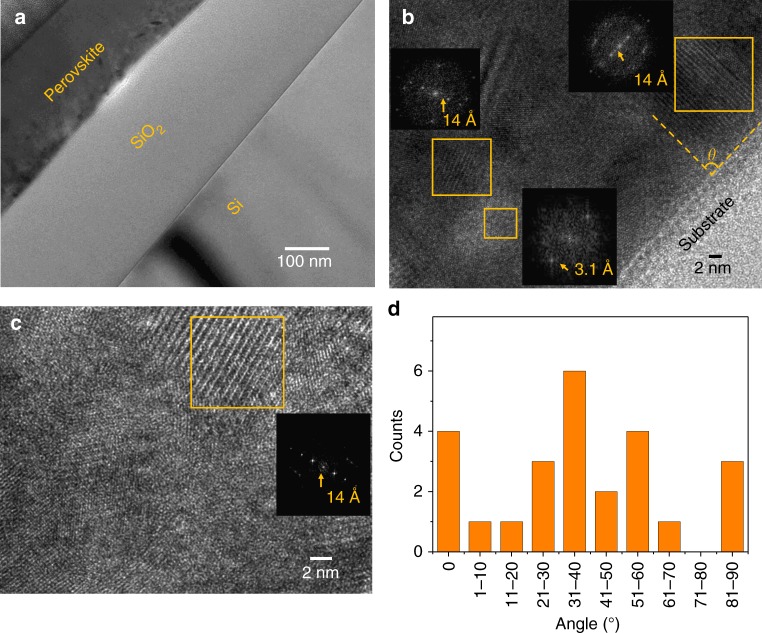
High-resolution transmission electron microscope (HRTEM) images of cross-section of the spin-coated BA_2_MA_3_Pb_4_I_13_ thin films. **a**–**c** TEM images of the cross-section of the thin film at different locations and different magnifications. Inset: images of the fast-Fourier transforms (FFT) of the area in yellow square. **d** Statistical distribution of the angle *θ* between substrate and layered perovskites

After identifying the nonuniform distribution of multiple layered perovskite phases in both vertical and lateral directions, we further studied their size and orientation with high-resolution transmission electron microscope (HRTEM). The HRTEM images, as well as associated fast-Fourier transforms (FFT), of the thin film’s cross-section shown in Fig. 4, convey a better picture of the overall morphology of the real layered films. In Fig. 4b, the 14 Å lattice spacing corresponds to the BA_2_PbI_4_ (*n* = 1) phase. Most of these layered domains have sizes in the range of several to tens of nanometers, much smaller than the film thickness. Surrounding the BA_2_PbI_4_ (*n* = 1), the narrower plane spacing of 3.1 Å indicates the presence of 3D MAPbI_3_. HRTEM allows us direct observation of the orientation of the layered perovskites. Very careful sample preparation utilizing the focused ion beam lift-out procedure is required to obtain a clear HRTEM image. Figure 4b, c shows two typical HRTEM cross-sectional images at locations close to and away from the substrates. To reveal the orientation distribution of the layered perovskite phases, we randomly chose 25 areas of layered perovskite phases for HRTEM study. The results are shown in Fig. 4d. Here, *θ* is the angle between the substrate and (001) planes of layered perovskites. Thus, 90˚ and 0˚ represent that the layered perovskite layer is perpendicular to and parallel with the substrate, respectively. From Fig. 4d, there might be apparent preferred orientation of the layered perovskite regions, but one would need more HRTEM images for better statistics.

### Model of growth kinetics and device operation mechanism

**Fig. 5:**
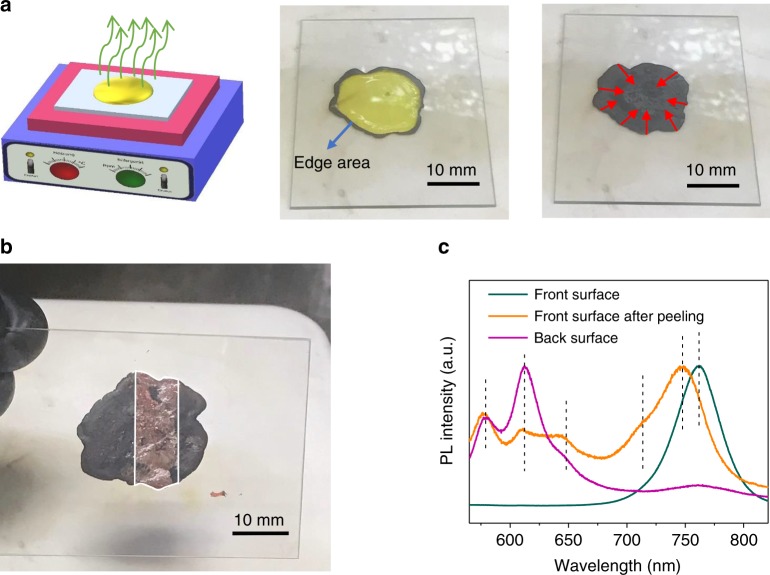
Optical images and photoluminescence (PL) spectra of top-crust peeling-off test. **a** Schematics of top-crust peeling-off test setup. A glass substrate (40 mm × 50 mm) with 100 µL BA_2_MA_3_Pb_4_I_13_ precursor solution (2 M in *N,N*-dimethylformamide (DMF)) on top was placed on a hot plate at 70 °C. The yellow solution begun to turn dark in the edge, and this dark area gradually spread into the center as indicated by the red arrows, forming a top-crust. **b** A Kapton tape was used to partially peel off the top-crust, and the revealed yellow precursor solution underneath that has not yet crystallized started to turn red under heating. After the solution had dried, the glass substrate was removed from the hot plate. The area inside the white frame represented the crystallized front surface revealed after the top-crust was removed by Kapton tape. **c** PL spectrum of different areas in **b**

A model of film formation mechanism is proposed to explain the observed morphology, i.e., the presence of both vertical and lateral phase segregation, and with most of layered domains with sizes in the range of several to tens of nanometers are surrounded by 3D-like perovskites. In the hot-casting process, hot precursor solution was casted on a hot spinning substrate. It is highly likely that the precursor solution at the liquid–air interface would reach supersaturation first due to the fast evaporation of DMF solvent at the surface, which has been confirmed by a top-crust scraping test^22^. As shown in Fig. 5a, b and Supplementary Movie 1, we find that 3D-like crystal tends to first precipitate prior to layered perovskites. This is a similar top-crust peeling-off test but using precursor material for layered perovskites of *n* = 4. The fact that black shell first formed proves that 3D-like crystals are more easily to form, otherwise the shell would be dark-red colored for *n* = 4 layered perovskites. As shown in Fig. 5c, a PL peak at 762 nm was probed for the formed shell, which can be assigned to 3D-like perovskites. The consumption of much less BA in top 3D-like perovskite layer actually pushes more BA toward the substrate side so that the ratio of BA to MA gradually increased in the leftover solution. The more BA-rich solution accelerates the layered perovskite nucleus formation and allows the formation of layered perovskites, though there is still a competition of 3D-like perovskites and layered perovskites. Actually PL emissions from bottom layer after peeling off the top-crust or excite from glass substrate side show the presence of layered perovskites with *n* = 2 to 4, a group of layered perovskites with much large *n* values and 3D-like (*n* → ∞) perovskites in the film crystallized from the leftover precursor solution underneath the top-crust.

This basically explains the observed vertical phase separation with more layered perovskite with smaller *n* towards the substrate site, and lateral phase separation in both micrometer and nanometer scales. This size of layered perovskite grains is limited by how fast the BA ligands can diffuse to the site for layered perovskite growth, and the competition of 3D-like and layered perovskites may interrupt the growth of large grain layered perovskites, as evidenced by the HRTEM images. The competition of 3D-like and layered perovskite formation also indicates that the film morphology should be very sensitive to the process condition such as temperature or solvent drying speed. This is again supported by the study of layered perovskite formation by slowing down the 3D perovskite formation by adding dimethyl sulfoxide (DMSO) or *N*-methyl-2-pyrrolidone (NMP) to the precursor solution. DMSO in precursor solution has been shown to promote the layered perovskite growth^23^, which is also observed here by the much strong PL emission from the layered perovskites in both front and back side of the films, and clear XRD peaks at low diffraction angle (Supplementary Figure 5, Supplementary Note 3). This basically can be explained by the slowed-down 3D perovskite formation by DMSO or NMP due to the formation of intermediate phases. Sargent and colleagues^21^ have reported the observation of intermediate phase in reduced-dimensional metal halide perovskites when DMSO/NMP is used in solutions. This might also arise from differences in the solubility of the cation (BA or MA) in the different solvents.

**Fig. 6:**
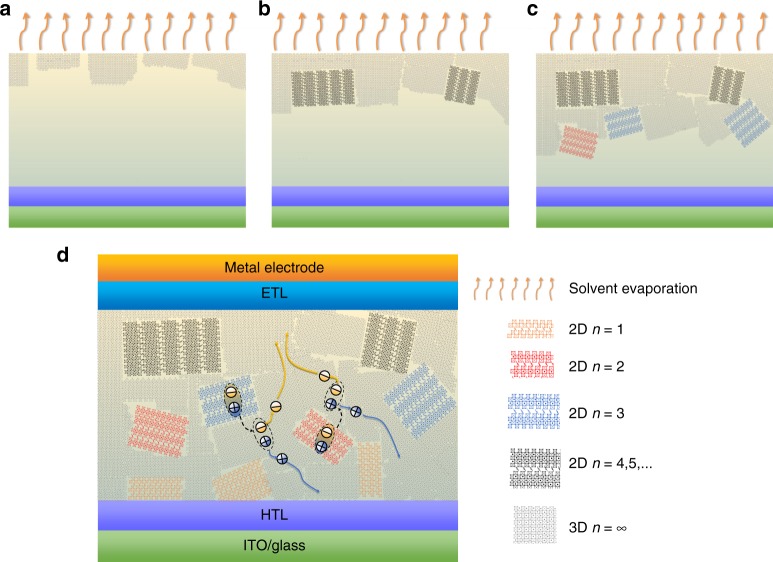
Growth kinetics, morphology and device operation mechanism model. **a**–**c** The growth kinetics and morphology model. **d** The device structure is indium tin oxide (ITO)/hole transport layer (HTL)/layered perovskite film/electron transport layer (ETL)/metal electrode

Based on the optical, XRD and HRTEM results discussed above, the growth kinetics and morphology of layered perovskite thin films fabricated by hot-casting is summarized in Fig. 6. The 3D-like crystal tends to precipitate first. As more and more 3D-like crystals precipitated, the ratio of BA to MA in leftover solution gradually increased and the BA-rich solution facilitated the afterward layered perovskite nucleus formation. As layered perovskite grains grew up, the BA/MA ratio in the surrounding solution gradually decreased and such MA-rich solution will again facilitate the 3D-like perovskite nucleation and growth, forming the morphology that layered perovskite grains with size of several to tens of nm were embedded in 3D-like perovskite network which agrees quite well with the atomic-scale morphology that we observed in HRTEM results. The spatial distribution of these layered perovskites was nonuniform in both vertical and lateral directions, and more layered perovskite phases with small *n* tend to segregate close to the substrate.

From the morphology scenario directly observed, we proposed a model for the operation of perovskite solar cells with layered perovskites (Fig. 6d): To contribute to the PCE of layered perovskite solar cells, the excitons generated in layered perovskite phases need to go through the layered/3D-like perovskite interface via a charge transfer or energy transfer. The transient absorption measurements were performed with the layered perovskite thin films (*n* = 4). The samples were excited from the back side (glass side) by a 20 μJ cm^−2^ 570 nm pump beam. As shown in Supplementary Figure 6, the TA signals with a negative sign around 572 nm, 604 nm, 639 nm and 704 nm are attributed to the resonance of *n* = 2, 3, 4, and 3D-like phase, respectively. For 3D-like and high *n* number 2D (*n* > 3) phases, the red-shift of the peak wavelength is caused by hot carrier cooling as the excitation energy is far above their bandgap. Growth of the 3D-like perovskite bleach peak and fast decay dynamics at 572 nm and 604 nm suggests an energy/charge transfer process between small *n* number 2D phase and 3D-like perovskite phase. According to previous studies^24,25^, we speculate that the energy transfer is more dominant in this system, because the transfer process happens at a very early delay time scale (far before 500 ps). We speculate energy transfer from the larger bandgap layered perovskites to lower bandgap 3D-like perovskites across the ligands. The layered flakes observed in our study were generally few layers, and thus we expect most of energy may be transferred to 3D-like perovskites. After energy transfer, excitons are generated in 3D-like perovskites, which will dissociate into free charge quickly due to the very small exciton binding energy in 3D-like perovskites. Thus, the transport and collection of free charge carriers was dominated by 3D-like perovskite phase which form a percolation network in layered perovskite solar cells.

### Carrier transport in layered perovskite solar cells

**Fig. 7:**
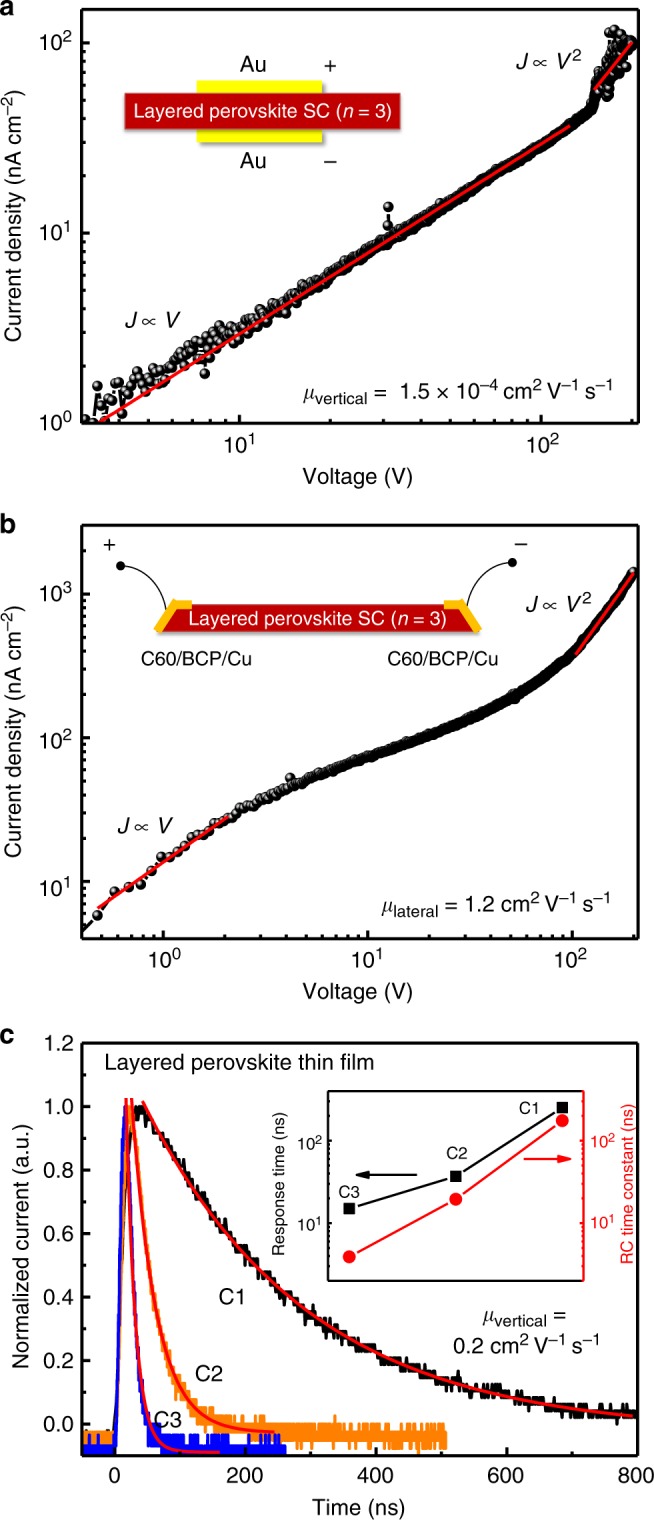
Carrier mobility study in layered perovskite single crystals and thin films. Current density-voltage curves for a hole-only vertical structured layered perovskite single crystal (SC) device (**a**) and an electron-only lateral-structured layered perovskite SC device (**b**). The insets show the device structure of vertical and lateral devices, respectively. **c** Transient photocurrent (TPC) curves of layered perovskite thin film solar cells with decreased working area (from C1 to C3). The inset is the measured RC time constant of the device as well as the fitted decay time of the TPC curves with different device working areas. The red lines are the fitting curves to the data

One way to verify whether photogenerated charges go through 3D-like perovskite phase to be collected is to measure the charge extraction time needed for both the 3D and layered perovskites with a thickness of the devices. In view of the layered structure of layered perovskites and the insulating nature of the long chain organic cations, it is intuitively assumed by us that there would be large conductivity anisotropy along the in-plane and out-of-plane directions. However, there is no study yet to find out whether the long organic ligands really inhibit the out-of-plane carrier transport properties. Here we used (BA)_2_(MA)_2_Pb_3_I_10_ (*n* = 3) single crystals (SCs) as a model system to measure the carrier mobility along these two directions with the space charge limited current (SCLC) method. Hole-only and electron-only devices were fabricated by sandwiching the layered perovskite SC between Au electrodes or C_60_/bathocuproine (BCP)/Cu electrodes, respectively, because of the large electron (hole) injection barrier at the layered perovskite/Au (C_60_/BCP/Cu) interface. For the out-of-plane mobility measurement, a gap larger than the crystal thickness is intentionally left between the electrode edge and the crystal edge when depositing the electrodes on the crystals, to avoid the influence of possible edge conductance of the crystal, as schematically shown in the inset of Fig. 7a. The device dark current density (*J*) as a function of bias (*V*) of the vertical structured hole-only device is shown in Fig. 7a, which can be divided into Ohmic response region ($J \propto V$) at the low bias, and the trap-filled SCLC region ($J \propto V^2$) under large bias. The *J*-*V* curve at the SCLC region can be well-fitted by the Mott–Gurney law:

1 $$J = \frac{{9\varepsilon \varepsilon _0\,\mu V^2}}{{8L^3}},$$

where *ε* is the relative dielectric constant that equals to 5 for (BA)_2_(MA)_2_Pb_3_I_10_, *ε*_0_ is the vacuum permittivity, *μ* is the carrier mobility and *L* is the distance between the electrodes. The hole mobility along the out-of-plane direction is fitted to be 1.5×10^–4^ cm^2^ V^−1^ s^−1^, which is comparable to carrier mobilities in organic semiconductors^26–28^. The in-plane electron mobility derived from the *J*-*V* curve of the lateral-structured electron-only device is approximately 1.2 cm^2^ V^−1^ s^−1^, which is nearly four orders of magnitude larger than that along the out-of-plane direction, and is smaller but close to that of 3D perovskites (Fig. 7b). For the electron-only vertically structured device, however, the *J*-*V* curve was dominated by the Ohmic region all the way up to the maximum bias applied, which made it difficult to derive the electron mobility along this direction. Nevertheless, the comparable conductivity at the Ohmic region for the hole-only and electron-only devices (Supplementary Figure 7) indicates that the electron mobility and hole mobility of (BA)_2_(MA)_2_Pb_3_I_10_ along the out-of-plane direction should be within the same order of magnitude.

The knowledge of the anisotropic mobility in layered perovskite crystals enabled us to determine the main charge transport path by measuring the carrier transit time in the layered perovskite thin films. Here, we used the transient photocurrent (TPC) method to measure the carrier transit time of the BA-based layered perovskite thin film solar cells (with precursor composition of *n* = 4) with a device structure of ITO/PTAA/layered perovskite (550 nm thick)/PCBM/C60/BCP/Cu, as shown in Fig. 7c. For the TPC measurement, the RC constant of the device was carefully controlled to be smaller than the carrier transit time by reducing device area. The derived low limit for the mobility of the layered perovskite films from TPC was 0.2 cm^2^ V^−1^ s^−1^ by neglecting the transit time in other buffer layers. This mobility is comparable to the in-plane carrier mobility of layered perovskite SC, or measured mobility of polycrystalline 3D perovskite materials^3,29^, but much larger than that of layered perovskites along the out-of-plane direction. Since we do not see layered flakes are large enough to connect electron and hole transport layers, the results agree well with our scenario that the charge extraction process in the layered perovskite thin films is dominated by the 3D-like perovskite phase.

## Discussion

The morphology observed in this study can help the understanding of the device efficiency limitation and stability enhancement in layered perovskite solar cells. The layered/3D-like mixture still allows the charge extraction out of the device through the 3D-like network if the film thickness is not too large. This generally explains the high EQE of 80 to 90% achieved in the wavelength range of 400 to 650 nm. However, compared to the EQE spectrum of pure 3D perovskite solar cells, it is found that EQE within the wavelength range of 650 to 750 nm is much lower for the layered perovskite solar cells. This may be caused by the inefficient absorption of the incident photons at this wavelength range, or due to the less efficient energy transfer from the layered phases with larger *n* values to the 3D-like phase because of either the larger flakes or the smaller bandgap difference between them. This presents a challenge to efficiently harvesting the incident photon energies for the layered perovskite solar cells, which is the main reason that the *J*_SC_ of the layered perovskite solar cells is lower than that of their 3D counterparts. Increasing the thickness of such films to enhance absorption generally does not always result in enhanced *J*_SC_, may be due to the fact that the layered perovskite flakes with non-ideal orientation embedded in 3D-like perovskite would still impede the charge transport and thus cause charge recombination. The *V*_OC_ of layered perovskite solar cells should be limited by the smallest bandgap phase when the photoactive layer is a mixture of multiple phases with different band gaps. This is the reason that the obtained *V*_OC_ of 1.13 V is comparable to the value widely achieved in 3D perovskite solar cells despite the layered perovskites has larger bandgap than their 3D counterpart. In terms of fill factor, it is much lower for the layered perovskite solar cells in comparison to the 3D counterparts, which may be caused by the increasing charge carrier recombination occurs at the layered/3D-like perovskite interface.

Regarding the better environmental stability of layered perovskite solar cells observed in some cases, it is generally believed that this is mainly benefited from the protection of the organic ligand of the layered perovskite materials. However, the real morphology result shown here reveals that the layered perovskite films formed by hot-casting method is mainly composed of 3D-like perovskite phases with more 3D-like phases preferentially distributed on the top surface. It is intriguing to understand why their long-term stability can outperform the 3D counterparts in some cases. We speculate the enhanced stability of perovskite solar cells with addition of some 2D additive may be contributed by multiple factors. First of all, the organic ligands added in the precursor solution for layered perovskite formation must stay somewhere if they do not convert all the 3D perovskites into layered perovskites. They most likely will stay at grain boundaries which may contribute to the formation of more robust grain boundaries. Our recent studies showed that defective grain boundaries may initiate and facilitate the film degradation, because moisture, oxygen or constituting ions in perovskites can migrate much faster along them^30^. As a matter of fact, our previous study on the ion migration property of the layered perovskite films fabricated by the same method as reported here showed that the ion migration was largely suppressed up to 330 K in layered perovskite films both in dark and under illumination^31,32^. This can significantly alleviate the ion migration-induced degradation of the perovskite layer, especially under illumination. Second, the addition of ligands for layered perovskites may also relieve the strain in the formed films and thus enhance the intrinsic stability of the films. The presence of lattice strain was recently observed by us in perovskite films formed through annealing process, and the lattice strain accelerates the degradation of perovskite films because ion migration in perovskite films is accelerated by the presence of strain^33^. The relief of strain can suppress the ion migration process and thus improve the photo-stability of MAPbI_3_ thin films. We propose that the better stability of layered perovskite solar cells may also have contribution from the less-strained perovskite layer in comparison to the pure 3D perovskite films, which is confirmed by the very small XRD peak shift between the layered perovskite film on substrate and the freestanding strain-free perovskite powders scratched from the substrate (see Supplementary Figure 8).

In summary, we conduct nanoscopic- and microscopic-level morphology characterization to reveal the real morphology of the hot-cast layered perovskite solar cells. Based on that, we propose a model to explain their working mechanism: the photons absorbed by the layered perovskite phases can contribute to the photocurrent by energy transfer process from the larger bandgap layered phases to the smaller bandgap 3D-like phase, and the carrier transport within the perovskite layer is mainly through the 3D-like phase which forms a percolating network across the vertical direction of the solar cell devices.

## Methods

### Preparation of perovskite solar cells

The ITO-coated glass substrates were cleaned sequentially in acetone and isopropyl alcohol under sonication for 30 min, twice for each step. After drying in a vacuum oven, the substrates were treated by ultraviolet (UV) ozone for 15 min and then transferred to nitrogen glovebox for use. The precursor solutions were prepared by dissolving specific stoichiometric quantities of BAI, MAI and PbI_2_ in DMF (molar ratio 2:3:4 for *n* = 4) with a concentration of 1.0 M. PTAA layers as the hole transport layer were firstly deposited on ITO substrates by spin-coating PTAA solution (0.2 wt.% in Toluene) at 6000 rpm for 35 s, followed by thermal annealing at 100 °C for 10 min. Then, the pre-heated PTAA-coated substrate was immediately transferred to the vacuum chunk of spin coater within 5 s, followed by dropping the hot perovskite precursor solution (60 μL, 70 °C) on hot substrate and spin-coating at 5000 rpm for 20 s without ramp. The hot-cast films were then annealed at 85 °C for 3 s. PCBM as the electron transport layers were directly deposited on top of perovskite layers by spin-coating the solution (20 mg mL^−1^ in 1,2-dichlorobenzene (DCB)) at 6000 rpm for 35 s and afterwards annealed at 100 °C for 30 min. C60 (buffer layers) and BCP (hole blocking layers) were thermally evaporated sequentially, which is followed by the thermal evaporation of Cu electrode.

### Film characterization

The XRD experiments were performed by a Bruker D8 Discover Diffractometer utilizing Cu Kα radiation. The SEM images were obtained with a Quanta 200 FEG ESEM scanning electron microscope. The absorption spectra were recorded by an Evolution 201/220 UV/vis (visible) Spectrophotometer. Photoluminescence measurements were performed with a Horiba 320 detector, and all the PL measurements were conducted in reflection mode. The transmission mapping of layered (BA)_2_MA_3_Pb_4_I_13_ thin films was taken by an optical microscope Olympus BX61 combined with a 650-nm long pass filter.

### Photovoltaic device characterization

The photocurrent density-voltage curves of the photovoltaic devices were recorded by a Keithley 2400 Source-Meter with homemade testing software. The devices were exposed to a xenon-lamp based solar simulator (Oriel 67005, 150 W) under AM 1.5 G irradiation (100 mW cm^−2^), the light intensity was calibrated by a Si photodiode (Hamamatsu S1133). The J-V tests were swept along forward direction with scan rate of 0.1 V s^−1^ and delay time of 100 ms. The EQE measurement was conducted using a Newport QE measurement kit, and the intensity of monochromatic beam of light was calibrated with a reference silicon photodiode and focused onto the working areas of devices.

### Synthesis of the layered perovskite single crystals

For layered perovskite single crystal with *n* = 3 synthesis, the liquid phase crystallization method was used as reported previously^11,34,35^. The 1.844 g lead(II) iodide, 198 μL *n*-butylamine and 0.477 g methylammonium iodide were completely dissolved in 6 mL hydriodic acid at 110 °C. Subsequently, the hot solution was slowly cooled down to room temperature, typically decreasing 10 °C for 1 h. Layered perovskite single crystal plate will gradually precipitate and grow at the solution–air interface.

### Characterization of the layered single crystal

The successful synthesis of layered perovskite single crystal with *n* = 3 was confirmed by the UV/vis absorption and XRD measurement. The Absorption spectra were recorded using an Evolution 201 UV/Visible Spectrophotometer. The X-ray diffraction patterns were obtained by a Rigaku D/Max-B X-ray diffractometer in the Bragg–Brentano parafocusing geometry. A conventional copper target X-ray tube equipped in the diffracted-beam monochromator was set to 45 kV and 40 mA.

### Grazing-incidence wide-angle x-ray scattering experiment

The GIWAXS patterns were measured at an incident angle of 1^o^ at beamline 7.3.3 at the Advanced Light Source in Lawrence Berkeley National Laboratory with a 10 keV X-ray energy on a Pilatus 2 M detector^36^. The patterns were corrected and produced using Igor Pro and a modified version of the NIKA package^37^. The color scale of the patterns is logarithmic. The molecular schematics in Supplementary Figure 3b and Supplementary Figure 3d were created using CrystalMaker® version 10.2.2. The layered perovskite (BA)_2_(MA)_3_Pb_4_I_13_ thin films for 2D GIWAXS measurement were fabricated with the same method as those in the high PCE layered perovskite solar cells, except that silicon (100) substrates were used while with PTAA layer on it.

### Transmission electron microscope characterization

The HRTEM samples were prepared by extracting the cross-section of the thin film fabricated by hot-casting. Transmission electron microscopy (TEM) lift-out samples were prepared via focused ion beam (FIB) polishing in a Helios NanoLab™ 660 SEM. The sample was extracted by FIB with accelerating voltage of 5 kV and current of 1.2 nA. After the lamella was prepared, it was welded to a TEM copper grid by platinum. After that, it was gradually thinned to electron transparency with an ion beam of accelerating voltage of 5 kV and gradually reducing current of 41 pA, 15 pA and 7 pA. The SEM accelerating voltage was kept at 5 kV for the entire process. Once the sample preparation was complete, the lift-out sample was transferred to the TEM chamber immediately in less than 1 min of exposure to air. The TEM was carried out by FEI Tecnai OsirisTM with 200 kV and 80 kV beam voltage.

To investigate if the samples are free from FIB damages, we doctor bladed the (BA)_2_MA_3_Pb_4_I_13_ precursor solution directly on copper grid. In Supplementary Figure 9, it can be seen that without FIB process the same wide lattice structure also showed up. Thus, the FIB process-induced damage can be excluded. Besides, damages from the transmitted electron beam were also investigated (in Supplementary Figure 10). We found that the sample transits from crystalline to amorphous phase when it is exposed to electron beam longer than 5 s^38^. Thus, all TEM images were taken within 5 s.

### Transient absorption spectroscopy

Transient absorption experiments are conducted with a 45 fs, 4 mJ Coherent Libra with a 1 kHz repetition rate. The 570 nm pump beam is from a visible continuum generated by focusing 1.5 mJ of the 800 nm fundamental into a 4 m long tube. An all-reflective 4 F setup is utilized to filter the desired portion of the spectrum. The spectrally filtered pump pulses have 5 nm widths and 250 fs durations. Continuum probe pulses are generated in a sapphire window and relayed to the sample with reflective optics. Signal detection is accomplished with a CMOS (complementary metal–oxide–semiconductor) array detector that is synchronized to the 1 kHz repetition rate of the laser system.

### TPC measurement

During this measurement, a nanosecond pulsed N_2_ laser was used to generate charge carriers close to the anode of the device that were driven toward the cathode by the built-in electric field, and the photocurrent was recorded by a GHz oscilloscope with an input resistor of 50 Ω. For the as-fabricated device with working area of about 8 mm^2^, the measured response time of the device was comparable to its RC time constant, i.e., 175 ns (Fig. 7c). This indicates that the intrinsic charge transit time across the device is smaller than the RC time constant, which is consistent with our previous reports. To eliminate the influence of RC time constant on the TPC signal, we gradually decreased the working area of the same device so that the device capacitance was reduced. It is found that the discrepancy between the device RC time constant and the TPC decay time is becoming larger when decreasing the device area, implying the charge transit time gradually dominates the TPC signal. Based on the response time of the smallest area device (*τ*), we can derive the carrier mobility in the vertical direction with the following expression:

2 $$\mu = \frac{{d^2}}{{V_{\mathrm{b}}\tau }},$$

where *d* is the film thickness and *V*_b_ is the built-in potential.

## Supplementary information

Supplementary Information

Description of Additional Supplementary Files

Supplementary Movie 1

## Data Availability

The data that support the plots within this paper are available from the corresponding author upon request.
