# Supplementary material for: Unveiling the operation mechanism of layered perovskite solar cells
Source: Nat Commun. 2019 Mar 1;10:1008. doi: 10.1038/s41467-019-08958-9 (PMC6397310; doi:10.1038/s41467-019-08958-9)
Supplement: Supplementary file 1 — Supplementary Information [file 41467_2019_8958_MOESM1_ESM.pdf]

Supplementary Information

Unveiling the Operation Mechanism of Layered Perovskite Solar Cells

Lin *et al.*

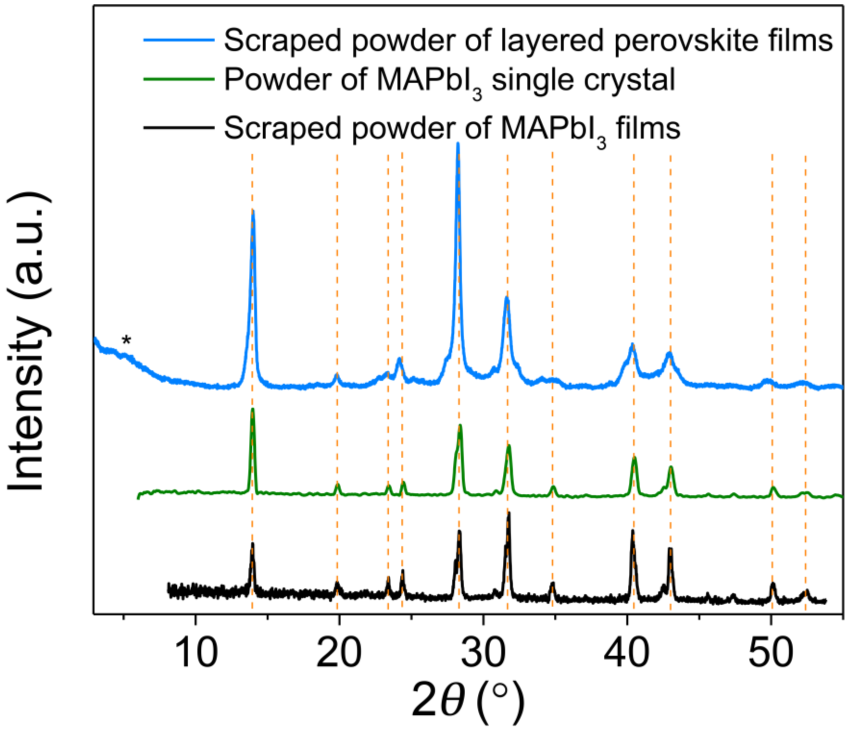

Supplementary Figure 1. Phase composition analysis of the hot-cast layered perovskite thin films with *n* = 4. XRD spectra of layered perovskite films scraped powder, $MAPbI_3$ films scraped powder and $MAPbI_3$ single crystal powder. (The green curve is adopted from ref. 1, and the black curve is adopted from ref. 2. Both of them are replotted to fit into the figure)

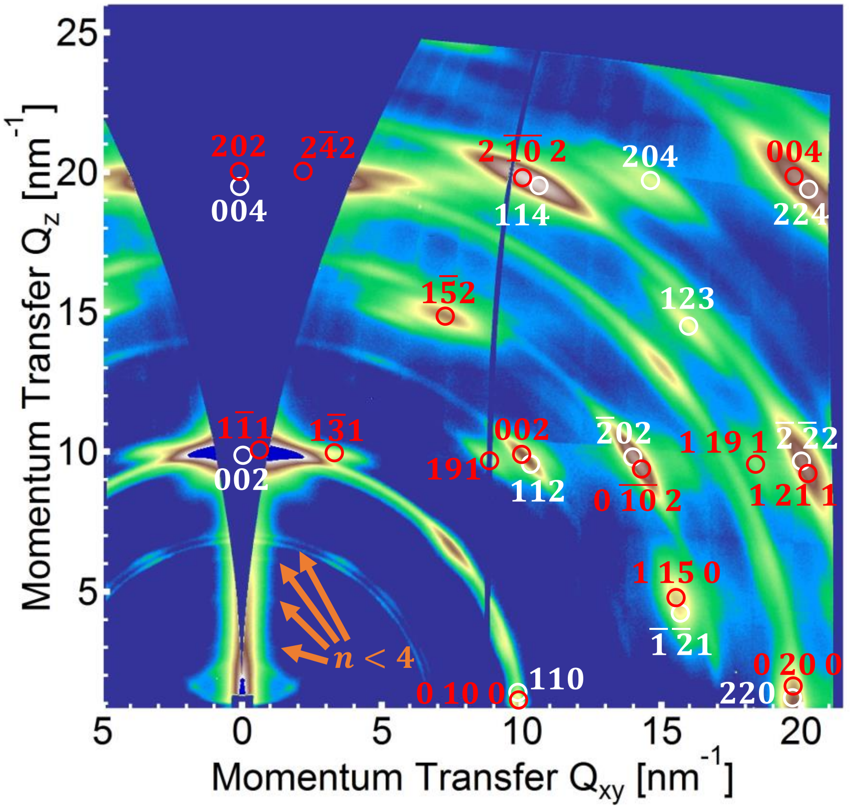

Supplementary Figure 2. GIWAXS pattern of the hot-cast layered perovskite thin films with *n* = 4. The (hkl) indices label the scattering peaks that can be accounted for by either a 3D perovskite (white) or a layered perovskite (red). The orange arrows indicate multiple low Q peaks, a distinct sign of several layered phases. The pattern is displayed in a logarithmic color scale.

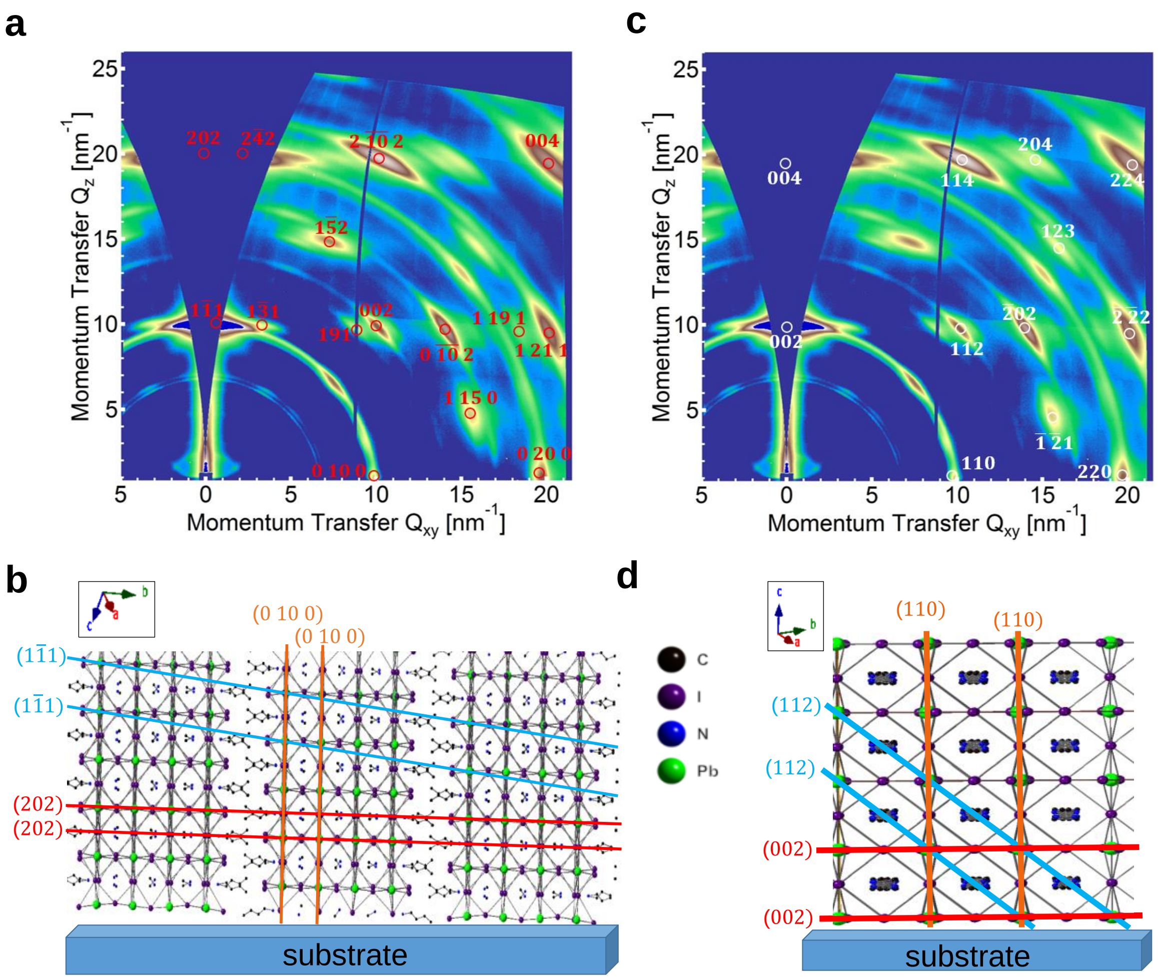

Supplementary Figure 3. GIWAXS pattern analysis of the hot-cast layered perovskite thin films with *n* = 4. a, GIWAXS pattern (displayed in a logarithmic color scale) with indexed peaks of a layered hybrid perovskites (*n* = 4). **b**, The schematic of the layered perovskites with respect to the substrate. **c**, GIWAXS pattern with indexed peaks of a 3D hybrid perovskites. **d**, The schematic of the 3D perovskites with respect to the substrate.

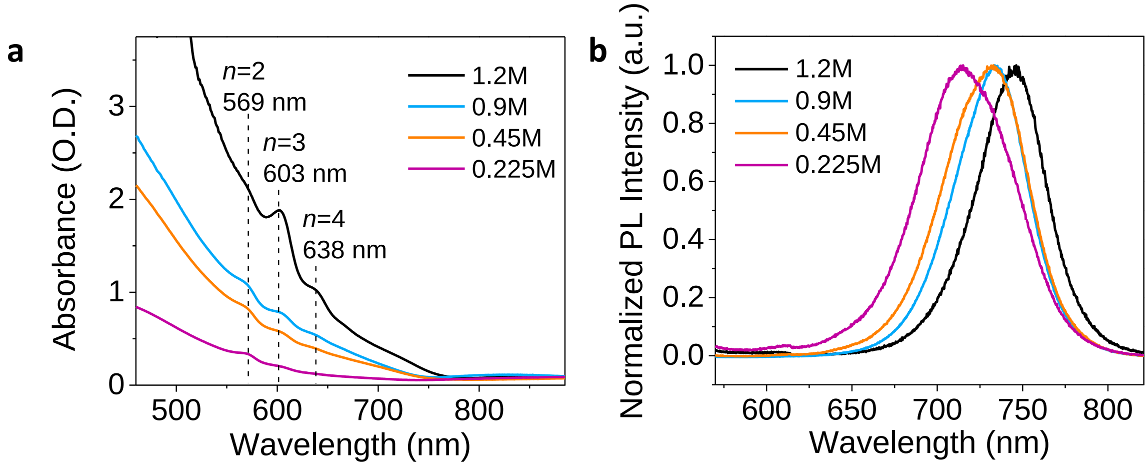

Supplementary Figure 4. Absorption and PL analysis of the hot-cast layered perovskite thin films with *n* = 4 prepared with molar concentrations of 1.2M, 0.9M, 0.45M and 0.225M. a, UV-vis absorption spectra of layered perovskite films. **b**, PL spectra of the films in **(a)** with the front-excitation.

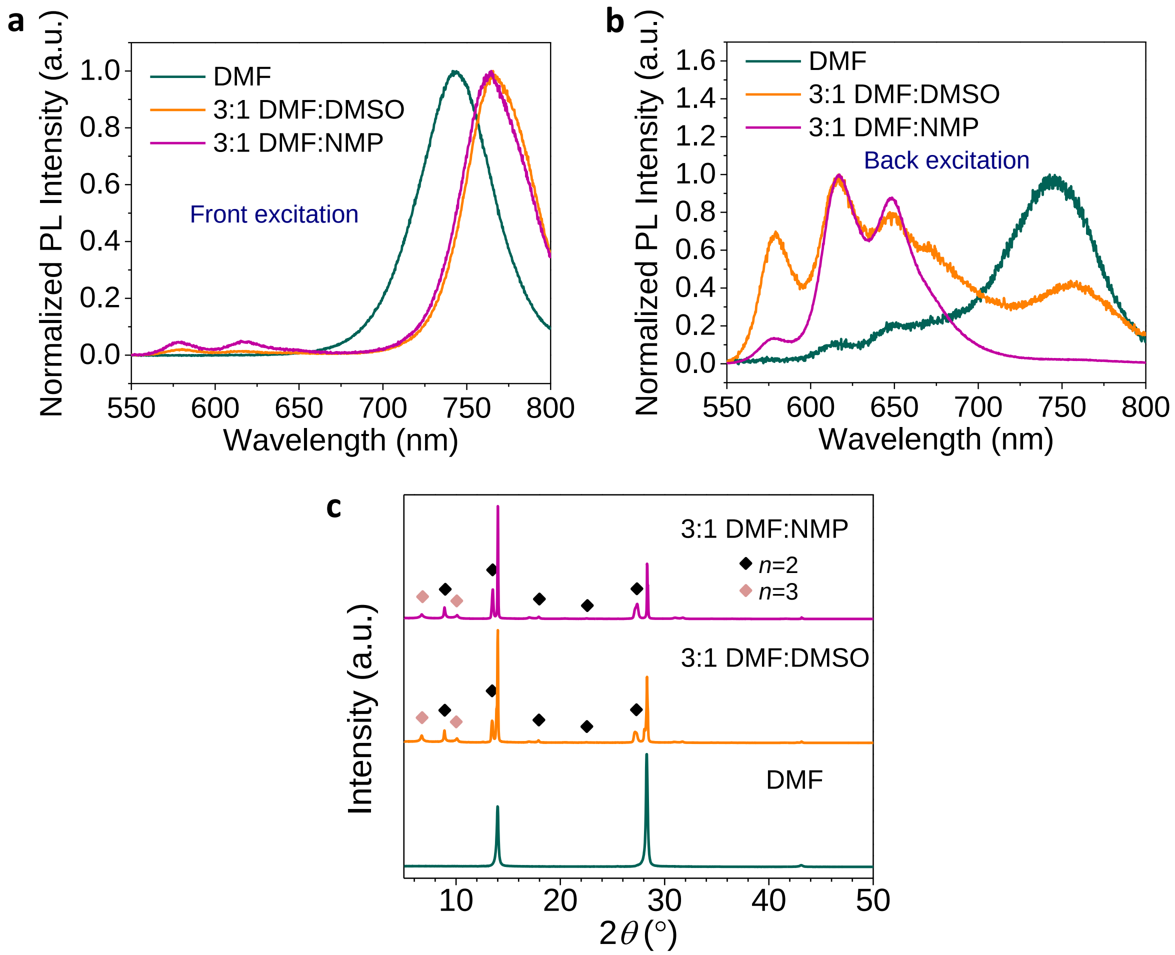

Supplementary Figure 5. Vertical and lateral phase segregation investigation. (a and b) The PL spectra of the hot-cast $BA_2MA_3Pb_4I_{13}$ films from precursor solutions using DMF, 3:1 DMF:DMSO, and 3:1 DMF:NMP illuminated from the front sides **(a)** and from the back sides **(b)** of the films. **c**, XRD pattern of the DMF, 3:1 DMF:DMSO, 3:1 DMF:NMP films (the pink dot indicates the diffraction peaks from {0k0} planes of $(BA)_2(MA)_2Pb_3I_{10}$ and the black dot indicates the diffraction peaks from {0k0} planes of $(BA)_2(MA)Pb_2I_7$.

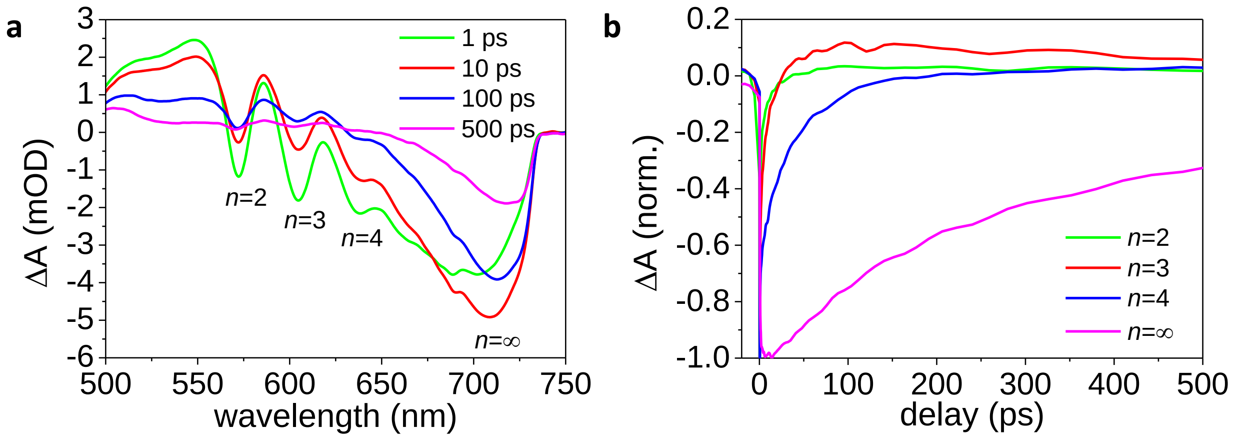

Supplementary Figure 6. Transient absorption (TA) results of the hot-cast layered perovskite thin films with 570nm pump wavelength. a, TA spectrum at 1 ps, 10 ps, 100 ps, 500 ps delay times. **b,** TA dynamics curve at the absorption wavelength of *n* = 2, 3, 4 and 3D-like phases.

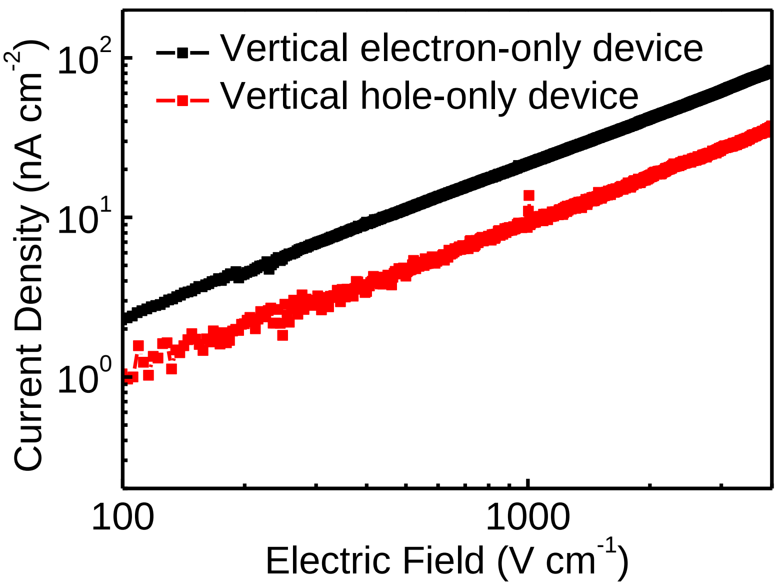

Supplementary Figure 7. Current density-electric field curves at the Ohmic region for the hole-only and electron-only $(BA)_2(MA)_2Pb_3I_{10}$ (n = 3) single crystal vertical devices.

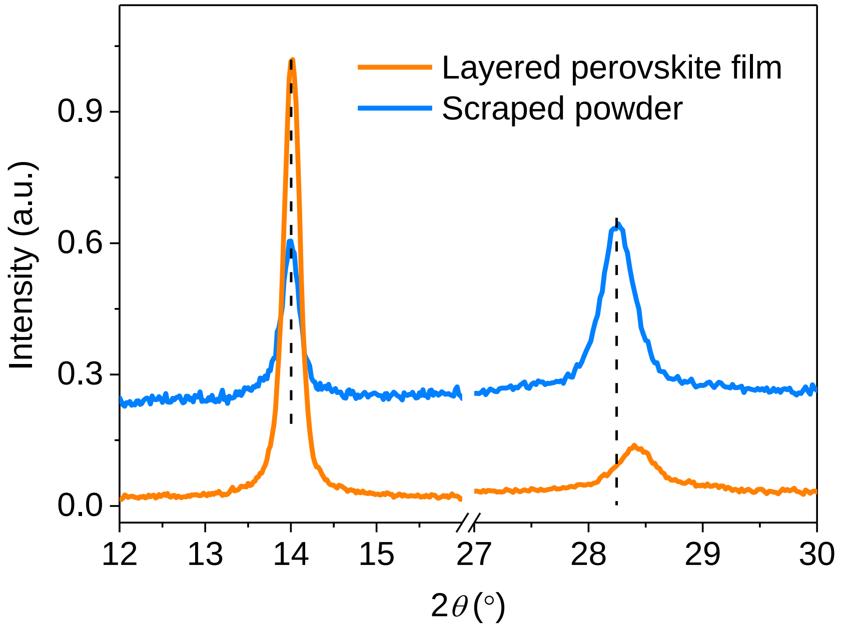

Supplementary Figure 8. Strain study of the hot-cast layered perovskite thin films with *n* =4. XRD spectra of the hot-cast layered perovskite thin films with *n* = 4 (orange curve) and powders scratched from the hot-cast layered perovskite thin films with *n* = 4 (blue curve). Compared to the XRD peaks of the free standing scraped layered perovskite powder, the XRD peak of the hot-cast layered perovskite thin film only at high angle shifted right, indicating a small out-of-plane compressive strain in the hot-cast layered perovskite thin film.

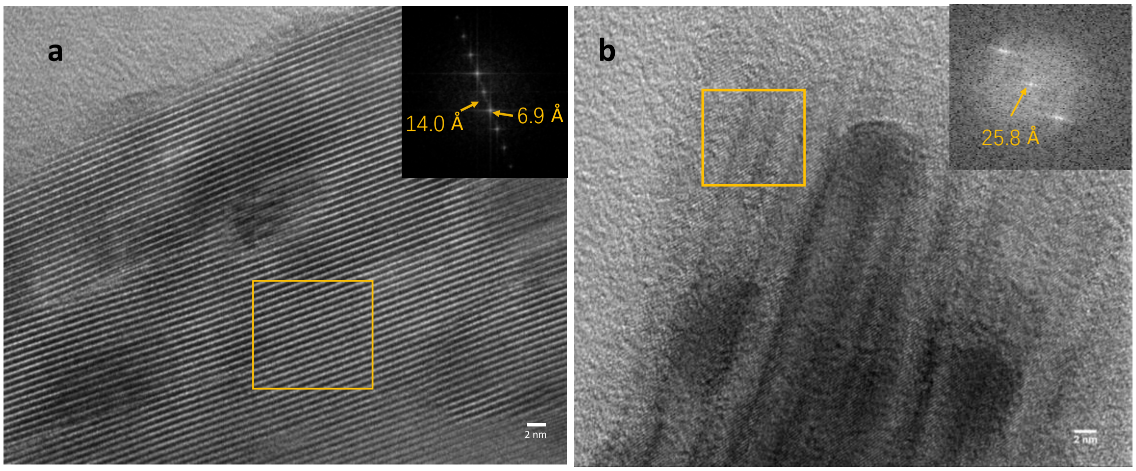

Supplementary Figure 9. TEM images of layered perovskite film on copper grid. In HRTEM images, beside the observation of BA_2PbI_4 (n = 1) phase (shown in Supplementary Figure 9a), the layered domains with lattice spacing of 25.8 Å were observed, which can be ascribed to the $BA_2MA_2Pb_3I_{10}$ (n = 3) phase. The layered domain was shown in the yellow square of Supplementary Figure 9b, and the associated fast-Fourier transforms (FFT) image was shown in the inset of Supplementary Figure 9b. Compared to BA_2PbI_4 (n = 1) phase, $BA_2MA_2Pb_3I_{10}$ (n = 3) phase seems less distinguishable from the surrounding 3D-like phase. This may make the observation of 2D domains with n values other than n = 1 more difficult. The HRTEM images in which $BA_2MA_2Pb_3I_{10}$ (n = 3) phase was surrounded by 3D-like phases can well support our proposed model for the spatial distribution of different phases in the thin film.

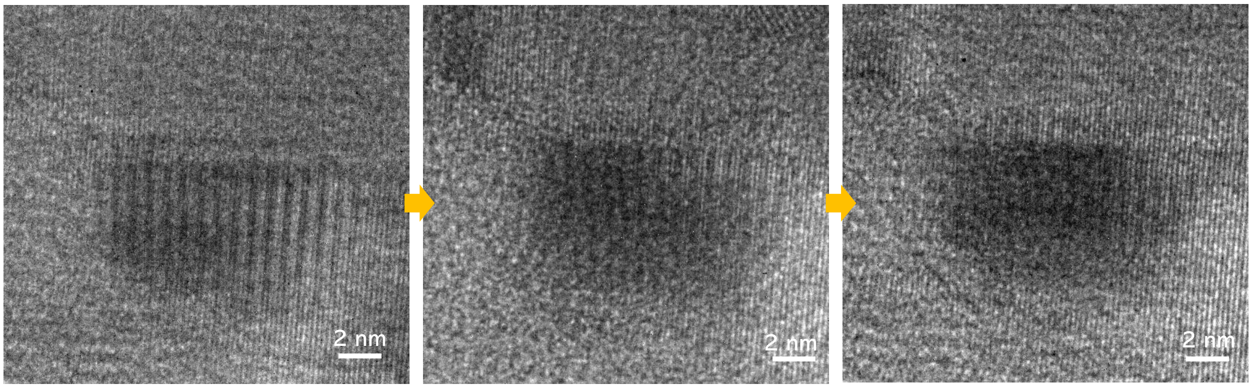

Supplementary Figure 10. **Damaged sample by TEM long time focusing.**

Supplementary Table 1. Calculated diffraction angles of layered perovskite $(BA)_2(MA)_{n-1}Pb_nI_{3n+1}$ (*n* = 2 to 4)

Compound	Theoretical $2\theta_{111}$/°	Theoretical $2\theta_{202}$/°	Theoretical $2\theta_{313}$/°
$(BA)_2(MA)Pb_2I_7$	14.2364	28.3306	43.1360
$(BA)_2(MA)_2Pb_3I_{10}$	14.1604	28.3314	43.1100
$(BA)_2(MA)_3Pb_4I_{13}$	14.1214	28.3250	43.0864

Supplementary Table 2. Analysis of the XRD data of $(BA)_2(MA)_2Pb_3I_{10}$ single crystals

2 θ/°	Calculated *d* pacing/Å	Indexed crystallographic plane	Theoretical interplanar spacing/ Å
7.072	12.4892	(040)	12.98975
10.465	8.446288	(060)	8.659833
13.871	6.379029	(080)	6.494875
17.303	5.120725	(0 10 0)	5.1959
20.735	4.280256	(0 12 0)	4.329917
24.193	3.675722	(0 14 0)	3.711357
27.677	3.220423	(0 16 0)	3.247438
31.187	2.865513	(0 18 0)	2.886611
34.723	2.581371	(0 20 0)	2.59795
38.298	2.348229	(0 22 0)	2.361773

Supplementary Table 3. The estimation of penetration depths for different incident angles of the excitation laser in layered perovskite thin films (*n* = 4)

Incident angle/ °	89	85	75	65	55	45
Penetration depth/ nm	57.7	57.8	58.7	60.4	62.6	64.9

Supplementary Note 1:

A Grazing-Incidence Wide-Angle X-Ray Scattering (GIWAXS) experiment was performed to investigate the phases and orientations of a “hot-cast” thin film of $(BA)_2(MA)_3Pb_4I_{13}$. With sharp and discrete Bragg peaks, the film generally exhibits crystalline nature as reported previously. [3] However, from rigorous GIWAXS analysis and peak simulation, the film does not appear to contain only one layered perovskite unit cell at one orientation; rather it consists of multiple phases and orientations. This is consistent with the findings of other experiments of this work[4-6] and leads to the proposed model, where a mixture of several phases is present. As an exercise to demonstrate this mixed nature, Supplementary Figure 2 also shows the white and red indices, highlighting the scattering peaks that agree with the simulated scattering peaks[7] for a layered[8] and a 3D[9] perovskite unit cell (at a certain orientation) respectively (see Supplementary Figure 3 for details). There exist many common peaks at the same Bragg position *e.g.* $(2\overline{1}\overline{0}2)$ from layered and (114) from the 3D phase, which demonstrates the difficulty in quantitatively determining the morphology here. A distinct peak, *e.g.* (191) from layered and (123) from 3D, can give hints that both layered and 3D perovskites are present at these orientations. Furthermore, as indicated by the few unidentified peaks, other phases are probably present as well besides these specifically analyzed here. For instance, the orange arrows indicate peaks that must be from other layered phases, [10] but do not belong to the set of white or red indices.

Supplementary Figure 3a and Figure 3c shows the GIWAXS pattern of a “hot-cast” thin film of $(BA)_2(MA)_3Pb_4I_{13}$. To demonstrate determining the phases and orientations in the film, indexGIXS[7] was used to analyze the GIWAXS pattern. With the input of unit cell parameters, indexGIXS helped to determine which of the unit cell planes could be parallel to the substrate. For a first analysis, unit cell information of $n = 4$[8] along with an orientation of the (202) plane parallel to the substrate was used (visualized in Supplementary Figure 3b). Supplementary Figure 3a shows the labeled scattering peaks that match the strong scattering* of the $n = 4$ unit cell. Sometimes two or three peaks with the same Bragg position were present; in such cases only one index was chosen for the figure for clarity. Clearly however, these indexed peaks are not sufficient to entitle the presence of (202) oriented $n = 4$ only. To investigate further, the unit cell of the 3D[9] hybrid perovskite $MAPbI_3$ oriented with the (002) plane parallel to the substrate (visualized in Supplementary Figure 3d) was input into indexGIXS as well. Supplementary Figure 3c shows the GIWAXS pattern but now with peaks marked that match the simulated peaks of the 3D unit cell only. Again, these indexed peaks are not sufficient to entitle the presence of the 3D phase by itself at this orientation. Altogether, this separate indexing of the same scattering pattern suggests that the film is made of layered and 3D-like phases, with the possibility of having more mixed phases and orientations as suggested by a few peaks that remain un-indexed in both cases.

*Strong scattering peaks were defined by the most intense peaks in simulated powder XRD of the unit cells in question. From a sample with a mixture of phases and/or orientations, one can expect a range of peak intensities in the GIWAXS experimental data, where weaker peaks might not be as visible as stronger scattering peaks. Therefore in this study weaker scattering peaks were not considered in simulating the scattering data. Crystallographic Information Files (CIFs) from literature (one of $n = 4$ and one of $MAPbI_3$[9]) were opened in CrystalMaker® 10.2.2 and subsequently simulated powder X-ray (using a wavelength of 1.24 Å to match the GIWAXS experiment) diffraction patterns were generated in CrystalDiffract® 6.7.3. The set of peaks were

selected based on the relative intensity, where the most intense 20 2D peaks and 14 3D peaks were kept for analysis to later feed into the indexGIXS peak-location simulation. Furthermore, one should note that the GIWAXS experimental setup of this study allowed to probe a momentum transfer, Q, up to 25 nm^{-1}. Thus indexGIXS peak-location simulation on the GIWAXS data was performed for Q values up to 25 nm^{-1}, matching the experimentally observed GIWAXS range.

Supplementary Note 2:

Supplementary Table 1 shows the calculated diffraction angles of layered perovskite $(BA)_2(MA)_{n-1}Pb_nI_{3n+1}$ (n = 2 to 4). The diffraction angles of layered perovskites can be calculated by using Bragg's law, $2dsin\theta = n\lambda$, where d is the interplanar distance of lattice planes in layered perovskite materials, θ is the scattering angle, where n is a positive integer and λ is the wavelength of incident wave. Here we take the wavelength of Cu K-alpha (λ=1.5418 Å) for calculation. The Crystal Data and Structure information for $(BA)_2(MA)_{n-1}Pb_nI_{3n+1}$ (n = 2 to 4) at 293 K can be found in previous publication.[8] The crystal system for layered perovskite $(BA)_2(MA)_{n-1}Pb_nI_{3n+1}$ (n = 2 to 4) is orthorhombic crystal system. So the interplanar distance d_{hkl} of lattice plane (hkl) for layered perovskites can be obtained by using the equation, $\frac{1}{d_{hkl}^2} = \frac{h^2}{a^2} + \frac{k^2}{b^2} + \frac{l^2}{c^2}$, where h, k, l are crystal plane indices, a, b, c are lattice parameters of layered perovskite materials.

Supplementary Table 2 shows the XRD pattern indexing of layered perovskite single crystals (see Fig. 2b). First, the diffraction angle θ can be directly read in XRD pattern in Fig. 2b. The d spacing values of layered perovskites can be calculated according to Bragg's law, $2dsin\theta = n\lambda$, where θ is the scattering angle, n is a positive integer and λ is the wavelength of incident wave. And the wavelength of Cu K-alpha (λ=1.5418 Å) was used for calculation here. The crystal system of layered perovskite $(BA)_2(MA)_2Pb_3I_{10}$ (n = 3) is orthorhombic crystal systems and the lattice parameters a, b, c are 8.9275 Å, 51.959 Å and 8.8777 Å, respectively.[8] The theoretical interplanar spacing for {0k0} planes of $(BA)_2(MA)_2Pb_3I_{10}$ can be obtained by using the equation, $\frac{1}{d_{(0k0)}^2} = \frac{k^2}{b^2}$. After compare the theoretical and experimental d spacing results, the layered perovskite single crystal we synthesized is determined to be $(BA)_2(MA)_2Pb_3I_{10}$.

Supplementary Table 3 shows the estimation of penetration depths for different incident angles of the excitation laser with wavelength of 405 nm in layered perovskite thin films with n = 4 (see Figure 3a-c). The refraction effect was taken into consideration in this estimation process by using Snell's law, $\frac{sin\theta_1}{sin\theta_2} = \frac{n_2}{n_1}$, with each θ as the angle measured from the normal of the boundary, n as the refractive index (which is unitless) of the respective medium. The value of n_1 (refractive index of air) at the wavelength of 405 nm is 1.00028250.[11] The values of n_2 (refractive index of layered perovskite thin films) and α_2 (absorption coefficient of layered perovskite thin films) at the wavelength of 405 nm are not available in previous reports, so we take n_2 of 1.7 and α_2 of 1.4×10^5 cm^{-1} at the close wavelength of about 428 nm reported in previous publication for this estimation.[3] The penetration depth δ_d of the excitation laser in layered perovskite thin films (n = 4) was estimated by using the equation, $\delta_d = \frac{1}{\alpha_2} cos\theta_2$.

Supplementary Note 3:

The influence of solvent on the morphology of the hot-cast layered perovskite films was verified by comparing PL spectrum and XRD patterns of the films by using different solvents. The PL spectra of the hot-cast $BA_2MA_3Pb_4I_{13}$ films illuminated from the front sides and the back sides were shown in Supplementary Figure 5a and Supplementary Figure 5b, respectively. The PL emission with the lowest energy showed a red-shift for both 3:1 DMF:DMSO and 3:1 DMF:NMP films under front excitation, as shown in Supplementary Figure 5a, which may be due to the larger $MAPbI_3$ grains formed in these solvent systems. For back excitation (Supplementary Figure 5b), 3:1 DMF:DMSO and 3:1 DMF:NMP films showed stronger PL emission from layered perovskites with n = 2 to 5 than PL emission from $MAPbI_3$, indicating that more layered perovskites were formed. The XRD pattern of 3:1 DMF:DMSO and 3:1 DMF:NMP films also showed obvious diffraction peaks from {0k0} planes of $(BA)_2(MA)_{n-1}Pb_nI_{3n+1}$ with n = 2 to 3, which were negligible in the DMF films, agreeing with the PL study results (Supplementary Figure 5c).

Supplementary References

1. Chen, Z. *et al*. Thin single crystal perovskite solar cells to harvest below-bandgap light absorption. *Nat. Commun.* **8**, 1890 (2017).

2. Zhao, J. *et al*. Strained hybrid perovskite thin films and their impact on the intrinsic stability of perovskite solar cells. *Sci. Adv.* **3**, eaao5616 (2017).

3. Tsai, H. *et al.* High-efficiency two-dimensional Ruddlesden-Popper perovskite solar cells. *Nature* **536**, 312-316 (2016).

4. Wang, Z. *et al.* Efficient ambient-air-stable solar cells with 2D-3D heterostructured butylammonium-caesium-formamidinium lead halide perovskites. *Nat. Energy* **2**, 17135 (2017).

5. Liu, J. *et al.* Observation of Internal Photoinduced Electron and Hole Separation in Hybrid Two-Dimentional Perovskite Films. *J. Am. Chem. Soc.* **139**, 1432-1435 (2017).

6. Xing, G. *et al.* Transcending the slow bimolecular recombination in lead-halide perovskites for electroluminescence. *Nat. Commun.* **8**, 14558 (2017).

7. Smilgies, D.M. *et al.* Indexation scheme for oriented molecular thin films studied with grazing-incidence reciprocal-space mapping. *J. Appl. Cryst.* **40**, 716-718 (2007).

8. Stoumpos, C. C. *et al.* Ruddlesden-Popper Hybrid Lead Iodide Perovskite 2D Homologous Semiconductors. *Chem. Mater.* **28**, 2852-2867 (2016).

9. Yamada, Y. *et al.* Dynamic Optical Properties of $CH_3NH_3PbI_3$ Single Crystals As Revealed by One- and Two-Photon Excited Photoluminescence Measurements. *J. Am. Chem. Soc.* **137(33)**, 10456-10459 (2015).

10. Venkatesan, N. R., Labram, J. G. & Chabinyc, M. L. Charge-Carrier Dynamics and Crystalline Texture of Layered Ruddlesden–Popper Hybrid Lead Iodide Perovskite Thin Films. *ACS Energy Letters* **3**, 380-386 (2018).

11. Ciddor, P.E. Refractive index of air new equations for the visible and near infrared. *Appl. Optics* **35**, 1566-1573 (1996).
