# Supplementary material for: Unveiling the operation mechanism of layered perovskite solar cells
Source: Nat Commun. 2019 Mar 1;10:1008. doi: 10.1038/s41467-019-08958-9 (PMC6397310; doi:10.1038/s41467-019-08958-9)
Supplement: Supplementary file 2 — Description of Additional Supplementary Files [file 41467_2019_8958_MOESM2_ESM.pdf]

Description of Additional Supplementary Files

File Name: Supplementary Movie 1
Description: A top-crust peeling-off test displaying the crystallization process of precursor material for layered perovskites of n=4 as shown in Fig. 5a, b.
